# Modulation of Paracetamol-Induced Hepatotoxicity by Acute and Chronic Ethanol Consumption in Mice: A Study Pilot

**DOI:** 10.3390/toxics12120857

**Published:** 2024-11-27

**Authors:** Allan Cristian Gonçalves, Aline Meireles Coelho, Maria Laura da Cruz Castro, Renata Rebeca Pereira, Natalia Pereira da Silva Araújo, Flávia Monteiro Ferreira, Pedro Alves Machado Júnior, Sirlaine Pio, Camilo Elber Vital, Frank Silva Bezerra, André Talvani, William de Castro Borges, Emerson Cruz de Oliveira, Daniela Caldeira Costa

**Affiliations:** 1 Laboratory of Metabolic Biochemistry, Institute of Exact and Biological Sciences, UFOP, Ouro Preto 35402-136, MG, Brazil; allan.goncalves@aluno.ufop.edu.br (A.C.G.); alinemeireles0@yahoo.com.br (A.M.C.); maria.cruz@aluno.ufop.edu.br (M.L.d.C.C.); renatarp@ufop.edu.br (R.R.P.); nataliaop@gmail.com (N.P.d.S.A.); flavia.monteiro@aluno.ufop.edu.br (F.M.F.); 2 Laboratory of Experimental Pathophysiology, Institute of Exact and Biological Sciences, UFOP, Ouro Preto 35402-136, MG, Brazilfrank@ufop.edu.br (F.S.B.); 3 Laboratory of Immunobiology of Inflammation, Institute of Exact and Biological Sciences, UFOP, Ouro Preto 35400-000, MG, Brazil; sirlaine.silva@aluno.ufop.edu.br (S.P.); talvani@ufop.edu.br (A.T.); 4 Laboratory of Enzymology and Proteomics, Institute of Exact and Biological Sciences, UFOP, Ouro Preto 35402-136, MG, Brazil; 5 Laboratory of Exercise of Physiology, School of Physical Education, UFOP, Ouro Preto 35400-000, MG, Brazil; emerson@ufop.edu.br

**Keywords:** paracetamol, acetaminophen, ethanol, alcohol, oxidative stress, hepatotoxicity, liver damage

## Abstract

Paracetamol (APAP) overdose is the leading cause of drug-induced liver injury, leading to acute liver failure. However, the role of concurrent acute or chronic ethanol ingestion in this context requires further clarification. In this study, we investigated the effects of acute and chronic ethanol ingestion on APAP-induced hepatotoxicity. Male C57BL/6 mice were randomly allocated into four groups: control (C; water 2×/day for 7 days); APAP (single dose of APAP, 500 mg/kg); acute ethanol (AE; a single ethanol dose—10 mL/kg, and one hour later an overdose of APAP—500 mg/kg); chronic ethanol (CE; ethanol—10 mL/kg, 2×/day for 7 days; and on the last day, an overdose of APAP—500 mg/kg). The results showed that AE induced heightened liver damage, increased necrotic area, and elevated levels of ALT, AST, TBARS, and oxidized glutathione compared to the control group. The AE group exhibited diminished glutathione availability and elevated CYP2E1 levels compared to the other groups. CE maintained a hepatic profile similar to that of the control group in terms of necrosis index, ALT and AST levels, GSH/GSSG ratio, and CYP2E1 activity, along with the upregulation of gene expression of the glucuronidation enzyme compared to the APAP group. Proteomic analysis revealed that the AE protein profile closely resembled that of the APAP group, whereas the C and CE groups were clustered together. In conclusion, ethanol consumption differentially modulated APAP overdose-induced liver damage. Acute consumption exacerbated hepatotoxicity, similar to an APAP overdose alone, whereas chronic consumption appeared to mitigate this injury, at least within the parameters assessed in this study.

## 1. Introduction

Paracetamol (N-acetyl-p-aminophenol, acetaminophen, APAP), available since the 1950s, has become one of the most widely used analgesics and antipyretics worldwide [1]. Although considered safe at therapeutic levels (4 g/day or less), when used in doses higher than therapeutic levels, it can cause serious liver damage, potentially progressing to acute liver failure (ALF) [2,3]. At therapeutic doses, most APAP is metabolized via the glucuronidation and sulfation pathways and subsequently excreted in the urine. The remaining APAP is converted into a potentially hepatotoxic metabolite, N-acetyl-p-benzoquinone imine (NAPQI), by cytochrome P450 enzymes, especially CYP2E1. The formed NAPQI is then reduced by glutathione to non-toxic, soluble mercapturic acid, which is also excreted in the urine. In cases of APAP overdose, the glucuronidation and sulfation pathways become saturated, and the cytochrome P450 pathway produces excessive NAPQI, leading to the depletion of glutathione reserves, causing liver toxicity mediated by the formation of free radicals [4,5]. The concomitant use of different drugs can lead to an adverse drug interaction, resulting in a decrease in therapeutic efficacy or an increase in the rate of adverse effects. The consumption of ethanol, among other substances, is recognized as capable of causing such interactions by altering the pharmacodynamics or pharmacokinetics of medications when used simultaneously [6,7]. It is estimated that 2.3 billion people consume alcoholic beverages regularly (an average of 32.8 g of pure ethanol per day). Global per capita consumption increased between 1990 and 2017, and the World Health Organization (WHO) predicts an even greater increase by 2030. As the consumption of alcoholic beverages increases, there is a significant contribution to the increase in morbidity and mortality worldwide [8,9]. The liver metabolizes ethanol through three enzymatic pathways: alcohol dehydrogenase (ADH); cytochrome P450, in particular, by CYP2E1 (in this case, also called MEOS—microsomal ethanol oxidation system); and catalase (CAT). Class I alcohol dehydrogenase (ADH1) is considered the most important enzyme for ethanol metabolism. CYP2E1 and CAT are considered secondary pathways, while the other ADH classes (II, III, and IV) are considered less effective pathways [10]. Oxidative stress is believed to be an important factor in the development of alcoholic fatty liver lesions, and CYP2E1 activation is an important source of reactive oxygen species (ROS) in chronic alcoholic liver disease [11]. CYP2E1 is a microsomal enzyme that plays a crucial role in the metabolism of both ethanol and paracetamol. The interaction between these substances is known for its complexity, and the results of this interaction depend on certain factors, such as the dose administered and the duration of ethanol consumption [12]. Alcohol–drug interactions are common in individuals who regularly use paracetamol and/or are classified as frequent, heavy, or compulsive drinkers. In this context, although the medical community recognizes the interaction between paracetamol and ethanol, the underlying metabolic changes and short- and long-term toxic effects have not yet been completely understood [13]. Therefore, this study hypothesizes that acute and chronic ethanol consumption can modulate paracetamol-induced hepatotoxicity differently. Thus, the objective of this study was to evaluate metabolic outcomes in the liver of C57BL/6 mice through the combination of paracetamol overdose and acute and chronic ethanol consumption.

## 2. Materials and Methods

### 2.1. Animals and Ethical Care

To carry out this study, isogenic, adult male C57BL/6 mice were used, aged 8 to 12 weeks and weighing an average of 20 to 30 g. The animals were housed in polypropylene cages at the Animal Science Center of the Federal University of Ouro Preto (UFOP)—Brazil, with controlled temperature and humidity, subjected to 12-h light/dark cycles, access to water and food “ad libitum”. The experimental procedures were approved by the UFOP Animal Ethics Committee (CEUA) under protocol number 2899150322.

### 2.2. Experimental Design

The APAP-induced hepatotoxicity model in C57BL/6 mice has been previously standardized in our laboratory [14,15,16,17]. Janssen brand liquid paracetamol (100 mg/mL, batch: AT5616) was used at a dose of 500 mg/kg. The ethanol dosage used for mice was based on the work of [18,19]. The animals received a 50% ethanol solution (10 mL/kg). Before administering the last dose of ethanol and paracetamol, the diet was removed, and the animals remained fasting for 6 h to ensure gastric emptying.

The sample size was calculated using G*Power software version 3.1.9.2, with a power of 0.90 and an alpha value of 0.05. For the present study, the animals were randomly distributed into a control group (C) (n = 12) that received, via orogastric gavage, water for 7 days; the paracetamol (APAP) group (n = 15) received a single dose of 500 mg/kg of paracetamol via orogastric gavage; acute ethanol group (AE) (n = 15) received a 50% ethanol solution (10 mL/kg) in a single dose via orogastric gavage and, one hour later, a single dose of 500 mg/kg of paracetamol was administered; chronic ethanol (CE) group (n = 15) received by orogastric gavage a 50% ethanol solution (10 mL/kg) twice a day for seven days and, one hour after the last dose of ethanol, the single dose of 500 mg/kg of paracetamol. To maintain the animals under the same experimental conditions (gavage stress and handling), the APAP and AE groups were also subjected to orogastric water gavage twice a day during the 7 days of the experiment. The calculation of the volume of liquid gavage (water, ethanol solution, or paracetamol) was carried out using the average weight of the animals placed in the same cage.

**Figure 1:**
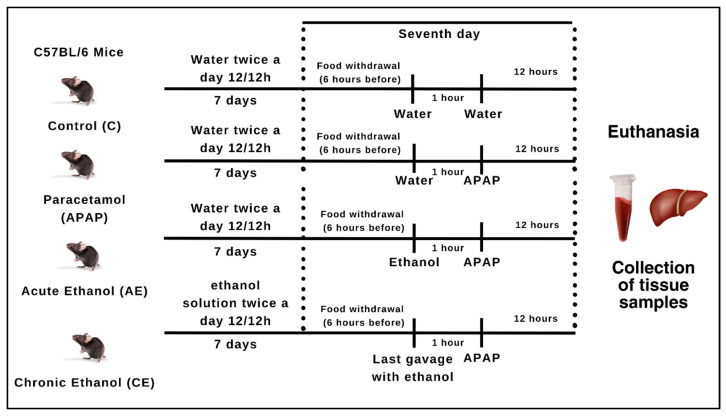
Experimental design. Source: Illustration created by the author himself through the website canva.com.

All animals were euthanized 12 h after administration of the paracetamol overdose. The animals were anesthetized by inhalation of isoflurane (Isoflurine, Cristália Ltd.a., São Paulo, Brazil, 1 mL/mL, lot: 21120059, 3–4% in air/O_2_ mixture). Then, exsanguination and collection of the animal’s blood and liver were carried out (Figure 1).

### 2.3. Serum Inflammatory Mediators

The concentration of inflammatory cytokines was determined in serum using commercial kits (PeproTech^®^, Rocky Hill, NJ, USA). The immunoabsorption assay was performed according to the manufacturer’s instructions using the kits described below: Murine IL-10 (catalog #900-K153) and Murine TNF-α (catalog #900-K54). Absorption readings were taken at 405 nm, allowing for wavelength correction, with adjustment to 630 nm.

### 2.4. Histological and Morphometric Analysis

Histopathological analyses of the liver parenchyma were carried out quantitatively and semi-quantitatively using morphometry and digital densitometry techniques through image scanning. The slides containing the liver sections were stained with hematoxylin-eosin and digitized with a 40× objective using a PRIMO STAR optical microscope coupled to an AXIOCAM 102 COLOR camera using ZEN^©^ Blue edition Software (version 3.3). Based on a normality test, 20 fields per animal were defined for each analysis. After obtaining the images with the 40× objective, we used the Image Pro Plus^©^ software—version 4.5.0.29 (Media cybernetics) for all analyses carried out. Histopathological analyses were carried out by a pathologist with experience in the field. The inflammatory nuclei present in the liver tissue were analyzed quantitatively by isolating the inflammatory nuclei through the application of contrast, saturation, dilation/erosion, and measurement of the nuclei’s area and automatic counting by the software Binucleated hepatocytes were counted manually in each image, taking as the criteria for binucleation the absence of space between the hepatocyte nuclei. The area of necrosis was assessed in all the images by manually measuring the extent of the necrotic area based on the morphology of the lesion, expressed in µm^2^/area. The presence of hepatic steatosis was assessed semi-quantitatively by applying a grid made up of 100 squares in which each square represented 1% of the area, and then evaluated according to a scoring system: (0) absent; (1) steatosis present between approximately 1–9%; (2) steatosis present between approximately 10–33%; (3) steatosis present between approximately 34–66%; (4) steatosis present between approximately 67–100% of the total tissue area [20,21].

### 2.5. Assessment of Liver Injury Biomarkers

#### 2.5.1. Measurement of ALT and AST Levels

To evaluate the activity of the hepatic enzymes alanine aminotransferase (ALT) and aspartate aminotransferase (AST), serum samples were used, and the analysis was carried out using commercial kits from the LABTEST^®^ laboratory (Lagoa Santa—Minas Gerais, Brazil), according to the protocols available by the manufacturer.

#### 2.5.2. Quantification of Thiobarbituric Acid Reactive Substances (TBARS)

To perform the TBARS dosage, 125 μL of trichloroacetic acid (TCA), 125 μL of thiobarbituric acid (TBA), and 62.5 μL of butylated hydroxytoluene (BHT) were added, according to a previously standardized protocol [14,15]. The reading was carried out on a plate reader with a wavelength of 535 nm. The straight-line equation was generated to calculate the concentration of the samples. The value obtained from the equation was divided by the protein content determined using the Lowry method [22].

#### 2.5.3. Carbonylated Protein

The quantification of carbonylated proteins was performed using a UV spectrophotometer at a wavelength of 370 nm, and the procedure was carried out by adding 10% TCA reagents, 2,4-dinitrophenylhydrazine (DNPH), ethanol/acetate, and SDS, followed by centrifugation and incubation at room temperature, protected from light, according to the protocol standardized by [14,15]. The results were expressed as nmol of incorporated DNPH/mL.

#### 2.5.4. Matrix Metalloproteinase 9 (MMP9) Activity

Matrix metalloproteinase 9 activity was quantified by zymography assay. Liver samples were homogenized using RIPA buffer (pH 8.0), which contained 150 mM NaCl, 1% IGEPAL^®^ CA-630 (Sigma-Aldrich^®^ Co., St Louis, MO, USA), 0.5% sodium deoxycholate, 0.1% SDS, and 50 mM Tris, and then centrifuged at 10,000× *g* for 10 min at 4 °C. Samples were prepared and inserted into polyacrylamide gels containing 8% gelatin (2 mg/mL). After running, the gels were washed in 2.5% Triton mM CaCl2 and 0.05% NaN3 (pH 7.5). The gels were stained using 0.05% Coomassie Brilliant blue G-250 for 3 h and then decolorized in 4% methanol with 8% acetic acid solution. Gelatinase activity was visualized as discolored bands against a blue background, indicating areas of proteolysis. The marked bands were quantified using Image J software version 1.32j (National Institutes of Health, Bethesda, MD, USA), where the optical density of each band was detected.

### 2.6. Assessment of the Antioxidant Profile

#### 2.6.1. Superoxide Dismutase (SOD) Activity

In the method used, the SOD enzyme competes with the superoxide radical, formed by the auto-oxidation of pyrogallol, responsible for the reduction of 3-[4,5-dimethylthiazol-2,5-diphenyltetrazolium] (MTT) to formazan crystals. Briefly, the plate was incubated in an oven at 37 °C for 5 min. The reaction was then stopped by adding 150 μL of dimethyl sulfoxide (DMSO), and the absorbance was measured at 570 nm using a plate reader.

#### 2.6.2. Catalase (CAT)

Liver homogenate samples were diluted in H_2_O_2_ (10 mM), and every 30 s, a spectrophotometer reading was taken at 240 nm to determine the absorbance of the samples until 2 min had been completed. For the blank, distilled water was used. Catalase activity was calculated using delta absorbance over 2 min. (final absorbance − initial absorbance/2) and the molar extinction coefficient of H_2_O_2_ (ε = 39.4 L/mol^−1^/cm^−1^). Therefore, 1 U of catalase was equivalent to the hydrolysis of 1 μmol of H_2_O_2_.

#### 2.6.3. Total Glutathione (GSHt) and Oxidized (GSSG) and Reduced (GSH) Fractions

The intracellular content of total glutathione (GSHt) and glutathione disulfide (GSSG) in the liver was determined using 5.5′dithio-bis (2-nitrobenzoic acid) (DTNB) and GSSG reductase (25). This assay employs a kinetic method based on the reduction of DTNB to TNB (5-thio-2-nitrobenzoic acid), which can be detected on a spectrophotometer at 412 nm.

For GSHt measurement, 10 μL of the supernatant was placed in a 96-well microplate. Then, 150 μL of the working mixture [95 mM phosphate buffer (pH 7.0), 0.95 mM EDTA, 48 μM NADPH, 0.031 mg/mL DTNB, 0.115 units/mL glutathione reductase were added. and 0.24% sulfosalicylic acid]. The samples were then incubated for 5 min at room temperature. The next step was to add 50 μL of NADPH (0.16 mg/mL). Absorbance readings were taken every minute for 5 min at 412 nm in an ELISA reader.

The procedure used to measure GSSG was the same as that adopted for total glutathione. However, the sample underwent a derivatization step by adding 2 μL of 2-vinyl pyridine. Derivatized samples were used in the assay following the same procedure described above. Absorbances were measured in serial dilutions of standard solutions of reduced and oxidized glutathione to generate calibration curves. The straight line equation was used to determine the concentrations in moles of total and oxidized glutathione per mL of sample. Reduced glutathione was obtained by subtracting the oxidized glutathione value from the total glutathione value.

#### 2.6.4. Quantification of Cytochrome P450 2E1 (CYP2E1)

The cytochrome P450 isoform 2E1 (CYP2E1) was quantified in the liver homogenate using the immunoenzymatic ELISA kit for mouse samples from the company ABclonal™ [Mouse Cytochrome P450 2E1 (CYP2E1) ELISA Kit]. CYP2E1-specific antibody was pre-coated on the microplate. Samples and standards were pipetted into the wells, and the CYP2E1 present bound to the immobilized antibody. Unbound substances were washed, and an antibody conjugate specific for CYP2E1 was added to the wells and bound to the CYP2E1 capture antibody combination in the sample. After a wash to remove any unbound antibody–enzyme combination, the conjugate was added to the wells. After the incubation and washing steps, a substrate solution was added to the wells. Absorbance was measured at 450 nm.

#### 2.6.5. Gene Expression Analysis by qRT-PCR

To analyze the expression of the genes under study, the quantitative post-reverse transcription polymerase chain reaction (qRT-PCR) technique was used. The concentration (measured at 260 nm) and purity (indicated by the 260/280 nm ratio) of total RNA were assessed using the NanoDrop ND1000 spectrophotometer (Nano Drop Technologies, Wilmington, DE, USA). The reactions were conducted in 96-well plates, with each well containing 1 μL of cDNA (diluted 5× in water), 0.5 μL of each primer (forward and reverse, 10 μM), and 5 μL of SYBR^®^ Green PCR Master Mix (Applied Biosystems^®^) and 3 μL of DNAse-free water, totaling a final volume of 10 μL. The assays were performed in technical triplicate for all genes evaluated, with the reference gene (GAPDH) present in all plates. The qRT-PCR reaction was conducted according to the programming contained in the ABI 7300 Applied Biosystems^®^ device. The specificity of the products obtained was confirmed by analyzing the dissociation curves of the amplified products at the end of each reaction. Expression level analyses were carried out using the relative gene expression quantification method (comparative Cq or ΔCq), which allows evaluation of the expression level of a specific gene between different samples. In this way, the expression of the target genes was normalized by the levels of the reference gene (GAPDH), and the results obtained through the difference between the expression of the target genes (ADH, CYP2E1, UGT1A1, and UGT2A1) and the expression of the gene of GAPDH reference. The sequence of the primers used was (1) ADH: F’ TAATGCCTCGGGGGACTTTG, R’ GAGAAGGTGCTGGTGCTGAT; (2) CYP2E1: F’ TTTCCCTAAGTATCCTCCGTGAC, R’ TCGTAATCGAAGCGTTTGTTG; (3) UGT1A1: F’ CTGGGAGGCTGTTAGTGTTCC, R’ GAGGCTTCAGGTGCTATGAC; (4) UGT2A1: F’ CTCATCTGGCCCATGGAAGG, R’ GGCCACAAGGACAGTCACAT; (5) GAPDH: F’ ATGTGTCCGTCGTGGATCTG, R’ GTAGCCCAAGATGCCCTTCA.

#### 2.6.6. Proteomic Analysis

Protein concentration was determined using the BCA protein assay kit (Thermo Scientific, Cramlington, UK). Fifty micrograms of protein were used for digestion. Samples were diluted in ammonium bicarbonate (AmBic, 25 mM) containing RapiGest (Waters Corporation, Milford, MA, USA) (0.06% final concentration), homogenized and incubated in a thermoblock at 80 °C for 10 min. Ten microliters of a dithiothreitol solution (DTT, Vetec, code: 13-1318-0001) were added, undergoing further homogenization and incubation for 10 min at 60 °C. The proteins were alkylated with the addition of 10 μL of an iodoacetamide solution (GE Healthcare UK Limited, code: RPM6302V) (33 mg/mL), homogenized again, and incubated for 45 min protected from light. Trypsin was added in a 50:1 ratio (protein: trypsin) followed by homogenization and incubated overnight (12 h). Next, 2 μL of peptides were separated using an UltiMate^®^ 3000 nanoUHPLC 3000 system (Dionex^®^), which was configured with an Acclaim PepMap100 C18 Nano-Trap capture column (75 μm i.d. × 2 cm, 3 μm, 100 Å; Thermo Scientific^®^) and an Easy Column C18 capillary column (75 μm i.d. × 10 cm, 3 μm, 120 Å; Thermo Scientific^®^) conditioned at 40 °C, at a flow rate of 300 nL/min. The peptides were previously washed with 2% acetonitrile and 0.05% trifluoroacetic acid for 5 min, then separated at 40 °C using a nonlinear solvent gradient A (0.1% formic acid) and B (80% acetonitrile/0.1% formic acid). Spectrum scans were acquired on the Q-Exactive™ mass spectrometry instrument (Thermo Scientific) coupled to the nano UHPLC system containing a nanoelectrospray ion source. The instrument operated at 3.5 kV, positive mode, resolution of 70,000 in the range 300–2000 *m*/*z*, maximum injection time of 120 ms, and automatic gain control (AGC) of 1 × 106 ions. Up to 12 most intense precursor ions with charge state ≥ 2 were isolated in a 2 *m*/*z* window and fragmented via high-energy collisional dissociation—HCD (30 V collision energy). MS/MS spectra were acquired at 35,000 resolution with a maximum injection time of 150 ms and AGC of 5 × 105 ions. Dynamic exclusion was set to 40 s. The .raw files obtained from the Q-Exactive instrument were searched in a database using Peaks Studio software, version 8.5. Protein identification was performed against a mus musculus database obtained from UniProt containing 55306 protein sequences. Relative protein quantification was performed and statistically tested using ANOVA, which was performed automatically by the software. Only proteins with a *p*-value ≤ 0.05 were considered differentially abundant between samples. The IDs of the researched proteins were used to search for the corresponding genes and the species, defined as *mus musculus*, in the database KEGG (Kyoto Encyclopedia of Genes and Genomes; http://bioinformatics.sdstate.edu/go/, accessed on 23 May 2024).

#### 2.6.7. Statistical Analyzes

Statistical analyses were performed using GraphPad Prism9^®^ software (GraphPad Software Inc., Solana Beach, CA, USA). Data normality was assessed using the Kolmogorov–Smirnov test. For data with a normal distribution, one-way ANOVA (analysis of variance) followed by the Bonferroni post-test was performed, and results were represented as the mean ± standard error of the mean. For data not following a normal distribution, the Kruskal–Wallis test followed by Dunn’s post-test was conducted, and results were represented as a median with an interquartile range. Differences were considered significant when *p* < 0.05.

## 3. Results

### 3.1. Assessment of Serum Inflammatory Profile

**Figure 2:**
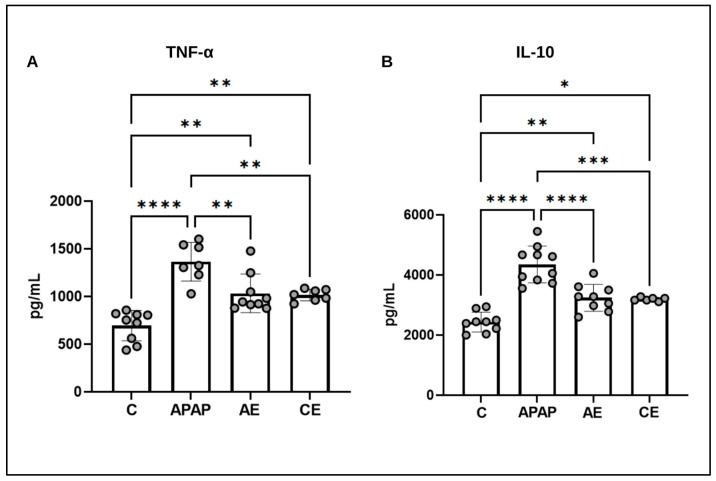
Assessment of the serum inflammatory profile using TNF-α (**A**) and IL-10 (**B**) measurements. Statistical analysis was performed using the one-way ANOVA test and Bonferroni post-test. TNF-α: Tumor necrosis factor alpha; IL-10: Interleukin 10. (*) represents a significant difference between groups. **** (*p* < 0.0001); *** (*p* < 0.001); ** (*p* < 0.01), and * (*p* < 0.05).

The serum inflammatory profile was assessed by measuring the levels of TNF-α and IL-10. Figure 2A shows that the APAP group had higher levels of TNF-α compared to the control group (*p* < 0.0001) and the AE (*p* = 0.0034) and CE (*p* = 0.0037) groups. The AE (*p* = 0.0013) and CE (*p* = 0.0047) groups were superior to the control group but equal to each other. A similar pattern was observed for IL-10 (Figure 2B), with the APAP group exhibiting higher values than the control group (*p* < 0.0001) and the AE (*p* < 0.0001) and CE (*p* = 0.0001). The AE (*p* = 0.0029) and CE (*p* = 0.0132) groups exhibited higher levels compared to the control group but did not differ from each other.

### 3.2. Histological and Morphometric Analysis

**Figure 3:**
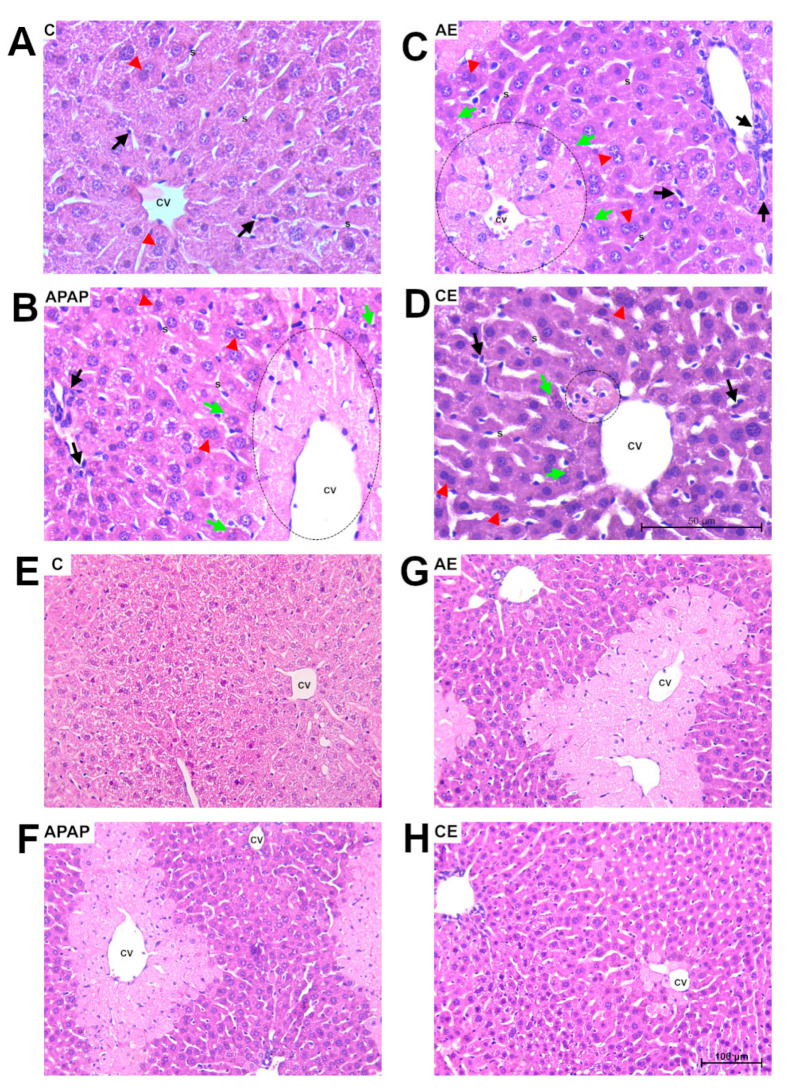
Histopathological aspects of the liver parenchyma. Photomicrographs of liver sections stained with hematoxylin and eosin. Bar = 50 μm, 400× magnification (**A**–**D**) and Bar = 100 μm, 200× magnification (**E**–**H**). (**A**,**E**) Representative image of the control group (**C**). (**B**,**F**) Representative image of the lesions found in the group treated with paracetamol (APAP). (**C**,**G**) Representative image of the group treated with acute ethanol (AE). (**D**,**H**) Representative image of the group treated with chronic ethanol (CE). In (**A**,**E**) preserved liver parenchyma, the presence of inflammatory cells in a normal pattern (black arrow), preserved sinusoidal capillaries (S), and few binucleated hepatocytes (red triangle). (**B**,**C**) Fatty degeneration (green arrow), binucleated hepatocytes (red triangle), area of extensive necrosis (dotted circle), and inflammatory infiltrates (black arrow). (**D**) Fatty degeneration (green arrow), binucleated hepatocytes (red triangle), and area of necrosis to a lesser extent (dotted circle), central vein (CV).

Representative images of the liver tissue are shown in Figure 3A–D at 40× magnification, highlighting the histopathological aspects of the liver parenchyma, and Figure 3E–H at 20× magnification, showing general pathological changes. The morphometric analyses conducted on the liver parenchyma of control animals (C) showed a preserved parenchyma without significant pathological aspects (Figure 3A,E). The other experimental groups presented: the presence of foci of inflammation in the APAP and AE groups (Figure 3B,C,F,G), a general increase in inflammatory nuclei, hydropic and fatty degeneration (Figure 3D), sinusoidal dilation, ischemic-type necrosis, with prevalent areas in zones 3 and 2, further away from the afferent blood supply, in addition to regenerative processes such as hepatocyte binucleation and karyomegaly.

**Figure 4:**
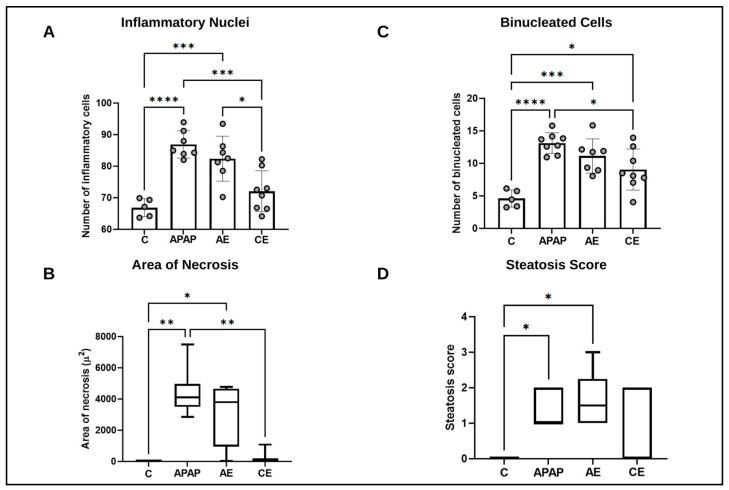
Morphometric and semiquantitative analyses of the liver in relation to inflammatory nuclei (Panel **A**), binucleated cells (Panel **B**), area of necrosis (Panel **C**), and steatosis score (Panel **D**). (**C**) Control group; (*APAP*) paracetamol group; (AE) acute ethanol group; (CE) chronic ethanol group. For results, number of inflammatory nuclei, number of binucleated cells, and area of necrosis, data were expressed as mean ± standard deviation of the mean and were analyzed by (one-way ANOVA) followed by the Bonferroni post-test. For the non-parametric results of necrosis area and steatosis score, data were expressed as the median and interquartile range (25th–75th percentile) and analyzed using the Kruskal–Wallis test followed by Dunn’s post hoc test. (*) represents a significant difference between groups. **** (*p* < 0.0001); *** (*p* < 0.001); ** (*p* < 0.01), and * (*p* < 0.05).

The inflammatory nucleus count was higher in the APAP and AE groups compared to the control group and CE group. APAP was higher than the control group (*p* < 0.0001) and CE (*p* = 0.0002). AE was greater than the control (*p* = 0.0006) and CE (*p* = 0.0095) (Figure 4A). Intoxication with paracetamol (APAP) and its association with an acute dose of ethanol (AE) promoted hepatocellular hypoxia. The APAP (*p* = 0.0033) and AE (*p* = 0.0456) groups showed a greater extent of the necrosis area compared to the control group, with no statistical difference between APAP and AE (*p* > 0.05). The APAP group presented a larger area of necrosis when compared to CE (*p* = 0.0073). The CE group was statistically equal to the AE and C groups (*p* > 0.05) (Figure 4B). Regarding the count of binucleated hepatocytes, the APAP and AE groups were statistically equal (*p* > 0.05). APAP was higher than the control group (*p* < 0.0001) and CE (*p* = 0.0117). AE was greater than the control (*p* = 0.0005) and equal to CE (Figure 4C). Fatty degeneration or steatosis was evident in a higher percentage in the APAP (*p* = 0.0309) and AE (*p* = 0.0152) groups compared to the control group. The CE group was not different from the control and, likewise, it was not different from the other groups (Figure 4D).

### 3.3. Assessment of Liver Injury Markers

**Figure 5:**
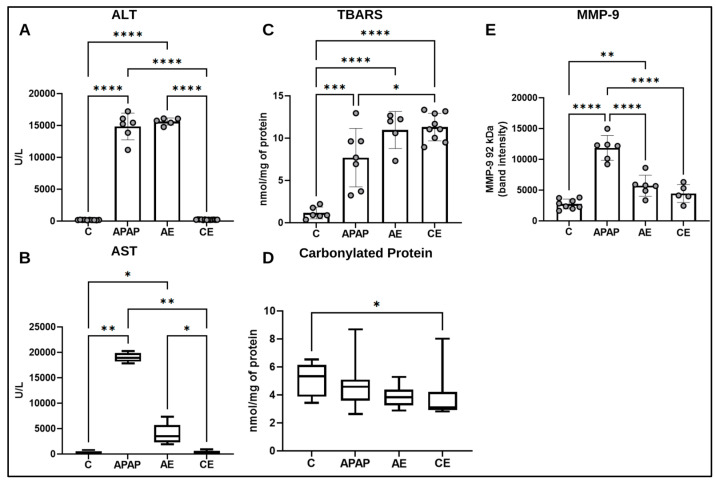
Assessment of markers of liver damage by evaluating the activities of ALT (**A**), AST (**B**), the concentration of TBARS (**C**), carbonylated protein (**D**), and the activity of MMP-9 (**E**). Statistical analysis for ALT, TBARS, and MMP-9 was performed using the one-way ANOVA test and Bonferroni post-test. For the statistical analysis of AST and carbonylated protein data, the Kruskal-Wallis test was applied, followed by the Dunns post-test. ALT: Alanine aminotransferase; AST: Aspartate aminotransferase; TBARS: Substances reactive to thiobarbituric acid; MMP-9: Matrix metalloproteinase—9. (*) represents a significant difference between groups. **** (*p* < 0.0001); *** (*p* < 0.001); ** (*p* < 0.01) and * (*p* < 0.05).

Regarding ALT activity (Figure 5A), there was a significant increase in the APAP (*p* < 0.0001) and AE (*p* < 0.0001) groups compared to the control group and the CE group (*p* < 0.0001 for both). The APAP and AE groups did not differ significantly. There was no difference between the CE group and the control group for this parameter. For AST activity (Figure 5B), there was greater activity in the APAP (*p* = 0.0017) and AE (*p* = 0.0472) groups compared to the control group. Similarly, the APAP (*p* = 0.0014) and AE (*p* = 0.0442) groups were higher than the CE group. The APAP and AE groups did not differ significantly from each other for this parameter, nor did the CE group differ from the control group.

Regarding TBARS, there was an increase in the APAP (*p* = 0.0001), AE, and CE (*p* < 0.0001 for both) groups compared to the control group. The AE and CE groups were not significantly different from each other; however, the CE group (*p* = 0.0226) was statistically higher than the APAP group. There was no significant difference between the APAP and AE groups (Figure 5C). As for carbonyl protein, the CE group (*p* = 0.0457) showed a significant reduction compared to the control group (Figure 5D). There was no statistical difference between the experimental groups for this parameter, nor was there any difference between the APAP and AE groups compared to the control group.

Regarding MMP-9 activity, the APAP group (*p* < 0.0001) showed the highest results compared to the control group and the AE and CE groups (*p* < 0.0001 for both). The AE group (*p* = 0.0093) showed a significant difference from the control group, which was not observed for the CE group (Figure 5E).

### 3.4. Assessment of the Hepatic Antioxidant Profile

**Figure 6:**
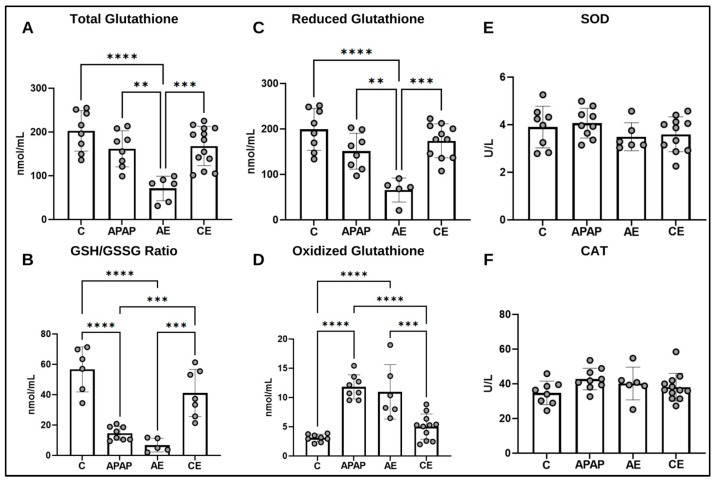
Assessment of the hepatic antioxidant profile by evaluating the levels of total glutathione (**A**), GSH/GSSG ratio (**B**), oxidized glutathione (**C**), reduced glutathione (**D**), SOD (**E**), and CAT (**F**). Statistical analysis was performed using the one-way ANOVA test and Bonferroni post-test. GSH: Reduced glutathione; GSSG: Oxidized glutathione; SOD: Superoxide dismutase; CAT: Catalase. (*) represents a significant difference between groups. **** (*p* < 0.0001); *** (*p* < 0.001), and ** (*p* < 0.01).

Total glutathione levels (Figure 6A) in the AE group exhibited the lowest values compared to the control (*p* < 0.0001), APAP (*p* = 0.0023), and CE (*p* = 0.0003) groups. The control, APAP, and CE groups were not significantly different from each other. Regarding the GSH/GSSG ratio (Figure 6B), the lowest values were observed for the AE and APAP groups (*p* < 0.0001 for both) compared to the control group. The APAP (*p* = 0.0009) and AE (*p* = 0.0002) groups were also smaller than CE. When evaluating the levels of reduced glutathione (Figure 6C), once again, the scenario presents itself with the lowest values for the AE group (*p* < 0.0001) compared to the control group. The AE group also has lower values than the APAP (*p* = 0.0038) and CE (*p* = 0.0001) groups. Finally, the results for oxidized glutathione (Figure 6D) show that the APAP group presented the highest levels for this parameter, being significantly higher than the control and CE groups (*p* < 0.0001 for both); however, it was no different from the AE group. The AE group, in turn, was higher than the control group (*p* < 0.0001) and CE (*p* = 0.0004).

Regarding the activity of the antioxidant enzymes SOD (Figure 6E) and CAT (Figure 6F), no significant differences were observed between the groups.

### 3.5. Assessment of CYP2E1 Levels and Expression of Genes Related to APAP and Ethanol Metabolism

**Figure 7:**
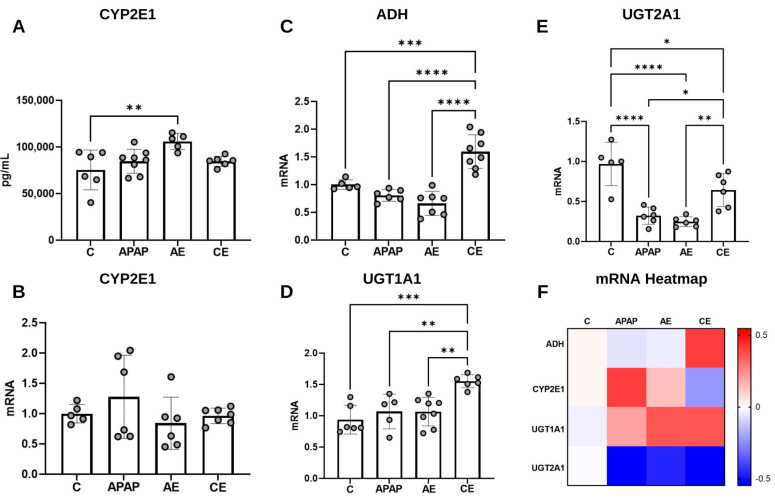
Assessment of hepatic CYP2E1 concentration (**A**), CYP2E1 gene expression (**B**), ADH (**C**), UGT1A1 (**D**), UGT2A1 (**E**) and gene expression heatmap (**F**). Statistical analysis for CYP2E1 concentration and expression of CYP2E1, ADH, and UGT2A1 genes was performed using the one-way ANOVA test and Bonferroni post-test. For statistical analysis of UGT1A1 gene expression data, the Kruskal–Wallis test was applied, followed by Dunn’s post-test. CYP2E1: cytochrome P450 2E1; ADH: Alcohol dehydrogenase; UGT1A1: Glucuronosyltransferase Family 1 Member A1; UGT2A1: Glucuronosyltransferase Family 2 Member A1. (*) represents a significant difference between groups. **** (*p* < 0.0001); *** (*p* < 0.001); ** (*p* < 0.01), and * (*p* < 0.05).

CYP2E1 is a microsomal enzyme active in both APAP and ethanol metabolism. When quantifying this enzyme (Figure 7A), it was found that the highest concentration values were found in the AE group, which was significantly higher in the control group (*p* = 0.0064), APAP (*p* = 0.0550) and CE (*p* = 0.0749). There was no significant difference in CYP2E1 gene expression between the experimental groups (Figure 7B).

ADH is an important enzyme in ethanol metabolism. Regarding gene expression for this enzyme, the CE group exhibited the highest values compared to the control group (*p* = 0.0004) and compared to the APAP and AE groups (*p* < 0.0001) (Figure 7C).

Regarding the expression of UGT1A1 (Figure 7D), the greatest difference was observed between the CE group (*p* = 0.0045) and the control group. The CE group also differed significantly from the APAP (*p* = 0.0071) and AE (*p* = 0.0024) groups. Finally, for UGT2A1 (Figure 7E), the APAP and AE groups presented the lowest values. These groups do not present statistical differences between them; however, they are significantly lower than the values of the control group (*p* < 0001 for both). The APAP group has lower results than the CE group (*p* = 0.0238), as well as the AE group (*p* = 0.0048). It can be considered that the CE group has intermediate values, lower than the control group (*p* = 0.0313) and, as already described, higher than the other experimental groups.

Figure 7F displays the heatmap graph referring to the qualitative analysis of the modulation of gene expression related to APAP/ethanol metabolizing enzymes. It is noted that in the APAP, AE, and CE groups, there is upregulation of the UGT1A1 gene and downregulation of the UGT2A1 gene. In the APAP and AE groups, upregulation of CYP2E1 was observed, while in the CE group, upregulation of the ADH gene was observed.

### 3.6. The Hepatic Proteome Is Altered During Acute and Chronic Alcohol Consumption and Acetaminophen Overdose

The scope of the proteome observed through spectral data obtained from the soluble fraction of the liver proteome was compared with the databases available for *mus musculus* animals at UniProt. Thus, it was possible to identify 937 distinct groups of proteins. Thus, it was possible to identify 937 distinct groups of proteins. Among the groups identified, 588 (62.75%) are present in all treatments. It was found that in the four experimental groups, there were groups of proteins that were expressed exclusively in each group (Supplementary Material—Figure S1). The dynamic range of identified proteins covered five orders of magnitude of difference between the highest and lowest abundance constituents in the liver, as assessed by the area under the curve. Around 200 proteins cover more than 90% of the accumulated area; among these, only 15 proteins correspond to more than 50% of the total ionic signal (Supplementary Material—Figure S2A,B). The 15 most abundant proteins are presented in decreasing order of abundance in Table S1 (Supplementary Materials—Table S1).

The analysis of proteomic data obtained from the soluble fraction of the liver proteome confirmed the presence of the proteins of interest that were quantified and evaluated through biochemical analyses in the present study. From the KEGG database, it was found that the genes for these proteins are involved in several drug and xenobiotic metabolism pathways via the cytochrome P450 pathway and glutathione metabolism, both involved in the metabolism and detoxification of paracetamol and ethanol (Supplementary Material—Figure S3).

**Figure 8:**
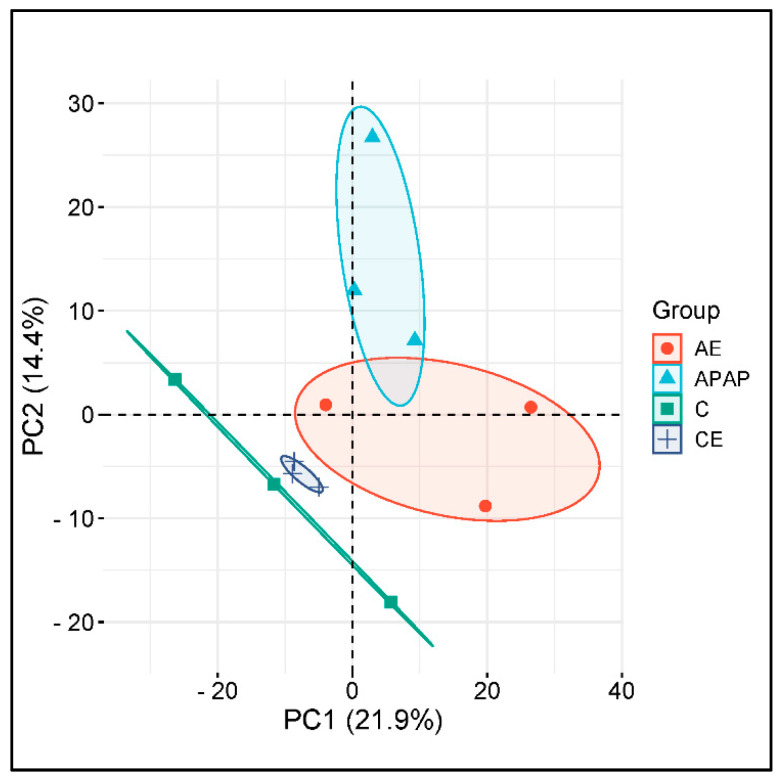
Principal components analysis (PCA). Axes demonstrate principal component 1 and principal component 2, showing 21.9% and 14.4% of the total variance, respectively.

Principal component analysis (PCA) demonstrated that the different experimental groups produced isolation or groupings of protein constituents according to the treatment used. The PCA graph revealed, according to the data from the label-free quantitative analysis, that the samples from the AE group were more closely related to those from the APAP group. Groups C and CE appear close to each other and further apart from groups APAP and AE. This analysis demonstrated that an acute dose of ethanol presents a profile of protein constituents closer to the acetaminophen overdose group (Figure 8), confirming the profile of the biochemical and morphometric analyses.

**Figure 9:**
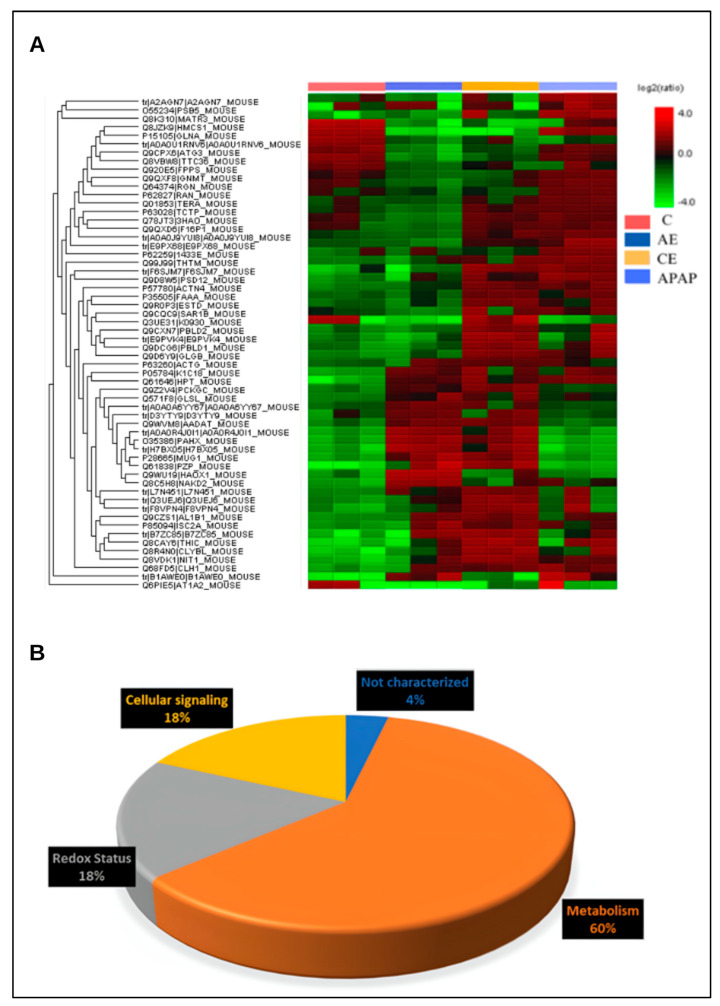
Heatmap of differentially abundant hepatic proteins when comparing different treatments. Red coloring indicates positive regulation, while green coloring indicates negative regulation (**A**) and classification of proteins according to their biological function (**B**).

The heatmap graph shows that among the most differentially abundant proteins, treatments with paracetamol and its association with an acute dose or chronic use of ethanol promoted positive regulation in several proteins. This result is likely associated with a compensatory mechanism aimed at maintaining cellular functions under the stress induced by the drugs (Figure 9A). Figure 9B shows the classification of proteins analyzed in the heatmap according to their biological function.

## 4. Discussion

The risk of APAP toxicity associated with alcohol consumption has been recognized since the 1980s; however, the mechanisms underlying liver injury are still not completely understood [23,24].

Our study was based on the premise that ethanol metabolism plays a critical role in modulating the extent of liver damage induced by paracetamol overdose. Consequently, the primary objective was to investigate the effects of co-exposure to ethanol and paracetamol. The 1-h interval between administrations was strategically chosen to maximize the interaction between the two compounds. This design allowed for the evaluation of co-exposure effects in both acute and chronic contexts, simulating clinical scenarios such as occasional alcohol consumption followed by analgesic use or repeated ethanol exposure accompanied by paracetamol intake.

Our results indicated that acute ethanol consumption maintained a hepatic profile similar to that of the group intoxicated with APAP. Although we observed a reduction in GSH levels beyond what was observed in the APAP group and an increase in CYP2E1 levels, the other parameters evaluated remained similar to those in the APAP group. This suggested that acute ethanol consumption does not worsen the hepatic response to APAP overdose. Chronic ethanol consumption improved the liver response to APAP. This was demonstrated by the reduction in inflammatory nuclei, area of necrosis, MMP-9, carbonylated protein, ALT, and AST, in addition to the increase in the GSH/GSSG ratio and gene expression of the enzymes UGT1A1 and UGT2A1, which are responsible for the non-toxic metabolism of APAP. Thus, it is suggested that, despite the observed increase in TBARS levels, chronic ethanol consumption minimizes APAP-induced hepatotoxicity. However, it is important to note that these results were observed after seven days of ethanol consumption; therefore, we cannot extrapolate these results to longer consumption.

During liver injury, exaggerated production of inflammatory cytokines can trigger a systemic inflammatory response syndrome. In this scenario, the analysis of serum cytokines is essential to understand and evaluate drug-induced liver failure [25]. TNF is a pleiotropic cytokine that can contribute to the induction of apoptosis and activation of endothelial cells and platelets, in addition to increased vascular permeability and activation of soluble mediators. The increase in TNF during the course of APAP-induced acute liver failure appears to be a crucial event in determining its outcome [26]. In our study, the APAP group exhibited the highest TNF values. Even with a lower dosage of APAP (300 mg/kg), it is possible to observe an increase in the levels of pro-inflammatory cytokines (TNF, IL-1β, and IL-6) 12 h after its administration [27]. Our results showed that the AE and CE groups had higher levels of TNF compared to the control group, although they were lower than the values in the APAP group. Chronic and acute ethanol consumption can increase TNF-production. This increase is attributed to an increase in the intestinal permeability caused by ethanol, which can lead to endotoxemia. Endotoxemia, in turn, results in the increased production of lipopolysaccharide, which activates Kupffer cells and ultimately leads to the generation of pro-inflammatory cytokines [28]. However, the deleterious effects of pro-inflammatory cytokines can be counterbalanced by the upregulation of immunoregulatory cytokines such as IL-10. IL-10 is an anti-inflammatory cytokine that is markedly released in cases of hepatotoxicity induced by both APAP and ethanol. IL-10 is capable of negatively regulating the synthesis of pro-inflammatory cytokines such as TNF [29]. Similar to the TNF results, higher levels of IL-10 were observed in the APAP group compared to the control group and the other experimental groups (AE and CE). However, the latter presented values that were lower than those observed in the APAP group but still higher than those in the control group. Even with a lower dose of APAP (300 mg/kg), a previous study [29] showed an increase in the serum concentration of IL-10, which peaked 24 h after drug administration. In another study, animals that received an ethanol dose of 5 g/kg three times at 12 h intervals also exhibited an increase in serum concentrations of IL-10 [30]. In contrast [31], using an ethanol dose of 7 g/kg in C57BL/6 mice, no changes were observed in TNF or IL-10 4 h after euthanasia. According to [31], in addition to the dose and the time of exposure, the serum concentrations of these cytokines can also be influenced by the strain of animals used. Therefore, C57BL/6 mice are less susceptible to changes in the systemic inflammatory response in models of excessive ethanol consumption.

After analyzing systemic TNF and IL-10 levels, we next evaluated histopathological changes in the liver. Morphometric analyses showed that treatment with APAP alone and in combination with an acute dose of ethanol (group AE) promoted hepatocellular hypoxia, a larger area of necrosis, and hepatic steatosis compared to groups C and CE. Similarly, both the APAP and AE groups exhibited higher counts of binucleated hepatocytes, which is evidence of tissue regeneration. A 300 mg/kg dose of APAP can promote necrosis and centrilobular liver degeneration [32]. Thus, the observed liver damage indicated a typical and consistent pattern caused by paracetamol overdose, which was maintained by acute ethanol consumption. The results of the histopathological analyses in the APAP group were consistent with those of previous studies conducted by our research group [14,15,16,17]. The literature suggests that when a combination of APAP (200 mg/kg) and ethanol [23] is used, following a protocol in which ethanol is administered via a liquid diet containing 5% ethanol (5% *v*/*v*) for 10 days, followed by ethanol gavage adjusted to the weight of the animals on the eleventh day (a longer period than that used in our study), the development of micro- and macroscopic hepatic steatosis was observed, in addition to hepatocellular ballooning. This indicated that ethanol administration aggravated APAP-induced liver injury.

Administration of 400 mg/kg of APAP is considered sufficient to cause significant liver toxicity within 6–24 h [33]. Even at a lower dose (300 mg/kg) than that used in our study, high plasma levels of ALT and AST were observed at different time points (3, 6, and 12 h) [32]. In previous studies (15–17), we administered a toxic dose of 500 mg/kg and performed euthanasia 12 h after drug administration, which revealed increased serum ALT and AST levels. An increase in ALT and AST activities was observed in C57BL/6 mice at a dose of 500 mg/kg, even 24 h after administration of the toxic dose [34]. Taken together, the results obtained from the different studies fall within the time window in which the ability of APAP to promote liver toxicity was observed (6–24 h). Consistent with these findings, in the present study, the APAP group showed higher ALT and AST levels. The AE group also showed higher serum activity of these enzymes compared to group C and similar to the APAP group, which was not observed in the CE group. Our data indicated that the combination of APAP overdose and acute ethanol administration promoted a greater degree of liver damage than chronic ethanol administration.

According to the literature, in the case of an APAP overdose, NAPQI can react even more with cellular proteins, increasing the formation of free radicals and lipid peroxidation and potentially worsening oxidative damage [35]. Similar to APAP, ethanol metabolism results in an increased production of free radicals, which play a crucial role in the development of liver damage caused by this substance. CYP2E1 is one of the three main enzymes involved in ethanol metabolism and is capable of converting it into more reactive products and increasing the generation of ROS [36,37]. Both acute and chronic ethanol administration can increase ROS production and lipid peroxidation in animal models and humans. Studies in which ethanol was administered via orogastric gavage showed that ethanol-induced liver injury was associated with increased lipid peroxidation and carbonyl protein formation and decreased hepatic antioxidant defense [18,28]. In both cases, free radical formation played an important role in inducing lipid, DNA, and protein damage [35,36]. Even in animals treated with ethanol ingested orally through a liquid diet (containing 36% of the calories from ethanol) for six weeks, the concentration of TBARS increased significantly, indicating oxidative damage to cellular lipids [37]. When evaluating the effects of acute ethanol administration (5 g/kg administered three times separated by 12 h), an almost nine-fold increase in hepatic TBARS content was observed in ethanol-treated animals compared to that in the control group [38], in which the concentration of TBARS was twice as high in animals chronically treated with ethanol via a liquid diet (Lieber–DeCarli). In our study, we observed an increase in lipid peroxidation as indicated by the TBARS levels in groups AE and CE, which were higher than those in groups C and APAP. Mice of the C57Bl/6J lineage were administered two doses of ethanol (6 g/kg) by orogastric gavage with an interval of 12 h between them [39]. As observed in the AE and CE groups, the cited study found an increase in the production of free radicals and lipid peroxidation through TBARS measurements. According to the authors, because the liver is the main site of ethanol metabolism, a reduction in hepatic proteasome activity by ethanol may have resulted in the formation of adducts or oxidative modifications of the proteasome subunit proteins when reacting with ethanol-derived acetaldehyde and/or secondary products (ROS and lipid peroxides) derived from ethanol oxidation.

The increase in TBARS levels in the APAP group was consistent with results obtained in previous studies conducted by our research group using the same APAP administration protocol [14,16]. These data demonstrated that the combination of APAP and ethanol intensified hepatic lipid peroxidation. Hepatic carbonylated proteins are considered important markers of APAP-induced oxidative stress [40]. In a previous study, no significant differences were found in this parameter between animals treated with ethanol and those in the control group [38]. However, regarding carbonylated proteins, we found that the APAP and AE groups showed no difference compared with the control group, and there was a reduction in this parameter in the CE group. Because protein carbonyls are markers of protein oxidation by ROS, the fact that the CE group presented the lowest values does not necessarily represent an inconsistency; rather, there was less protein damage caused by oxidative stress, which is also typical in cases of APAP overdose. This result does not necessarily represent a protective effect of ethanol and might be an adaptative mechanism of the organ to the oxidative insult caused by prolonged ethanol intake. However, given that the APAP and AE groups were not statistically different from the control group, we understand that the protein carbonylated assay protocol used in our study was probably less sensitive in assessing hepatic oxidative damage than the TBARS measurement.

MMP-9, also known as type IV collagenase (gelatinase B), belongs to a family of matrix zinc-dependent proteinases (MMPs), almost all of which play an important role in liver regeneration and in controlling the number of proteins in the extracellular matrix. They are also involved in other biological processes, such as fibrosis, cirrhosis, and carcinogenesis [41]. In our study, increased MMP-9 activity was observed in the APAP and AE groups. In another study, an increase in MMP-9 activity was observed 6 h after the administration of an APAP dose of 600 mg/kg [42]. These data suggest that an increase in MMP-9 activity resulting from APAP overdose is associated with liver injury. Sinusoidal endothelial cells (ESCs) are direct targets of APAP hepatotoxicity, as evidenced by the formation of clefts in the endothelium of centrilobular sinusoids formed by the destruction and/or coalescence of fenestrae. The formation of larger clefts in the sinusoidal endothelial cells results in increased infiltration of red blood cells into the extrasinusoidal space, causing additional hypoxic damage to the liver [43,44]. These events precede any evidence of histological or chemical injury. These cracks are larger and more frequent in animals treated with ethanol, acutely or chronically, after receiving a toxic dose of APAP. The inhibition of MMP-2 and MMP-9 can minimize endothelial injury and red blood cell infiltration, as they affect the ESC cytoskeleton, which, in turn, reduces cleft formation [43]. The increase in MMP-9 levels during APAP intoxication, as well as its association with acute ethanol administration, may be associated with hepatocellular damage and microcirculatory dysfunction, resulting in significant hepatocellular hypoxia, in line with the histological data from our study.

APAP toxicity is primarily associated with excessive production of NAPQI. Hepatic cytochrome P450 enzymes, particularly CYP2E1, play fundamental roles in the oxidative activation of APAP. In cases of APAP overdose, large amounts of NAPQI are generated and subsequently conjugated with glutathione (GSH), which can neutralize it [45]. The CYP2E1 pathway can also produce ROS, mainly superoxide anions and hydrogen peroxide, in alcohol-induced liver injury [46]. Both these mechanisms lead to mitochondrial dysfunction, resulting in increased ROS production, decreased ATP production, and compromised cellular antioxidant profiles [47]. Regarding the antioxidant profile, the AE group exhibited the lowest total glutathione values as well as the lowest GSH/GSSG ratio. The ICR mice were treated with APAP (400 mg/kg) or ethanol (4 g/kg) [48]. According to the authors, the administration of ethanol itself did not change the hepatic GSH and GSSG content; however, in animals that received combined APAP and ethanol, the GSH content decreased, although not significantly. However, both the APAP and ethanol dosages used by these authors were lower than those used in our study, and the animal strains used were different. In animals chronically treated with ethanol (5% *v*/*v*) and receiving an acute dose of APAP (200 mg/kg), the total glutathione values were the lowest among all groups studied. Finally, we found that both the AE and APAP groups presented the highest levels of oxidized glutathione. These findings suggested that the combination of acutely administered APAP and ethanol leads to a greater depletion of glutathione reserves, potentially resulting in increased liver damage due to the formation of protein adducts and underlying oxidative stress, ultimately leading to cellular collapse and necrosis.

Enzymes such as CYP2E1, alcohol dehydrogenase (ADH), and catalase (CAT) are involved in the oxidative pathway of hepatic metabolism. The microsomal respiratory chain and the CYP2E1-dependent microsomal monooxygenase system are the main sources of ROS during ethanol ingestion. When addressing the ability to produce diverse hepatotoxic substrates related to APAP and ethanol metabolism, CYP2E1 is of particular interest [49]. In the present study, group AE exhibited the highest levels of CYP2E1 compared to group C. However, when evaluating gene expression for the same parameter, we found no difference between the experimental groups. Taken together, these results suggested a temporal differentiation in the regulation of gene expression and protein levels. Thus, it is possible to infer that an increase in CYP2E1 gene expression in the AE group may have occurred more quickly, not allowing the detection of significant differences within 12 h, whereas only CYP2E1 protein levels were shown to increase after APAP overdose in the same time interval.

Chronic alcoholics are at greater risk of developing APAP-induced liver injury owing to the induction of CYP2E1 in the MEOS, which is also responsible for the production of NAPQI from APAP metabolism. Thus, it is assumed that high CYP2E1 activity results in greater conversion of APAP to NAPQI, thereby increasing APAP-induced liver toxicity [50,51]. Large-scale clinical studies on APAP poisoning have not observed a negative change in the prognosis due to prior ethanol ingestion [52]. However, when analyzing studies on APAP toxicity in combination with chronic ethanol use, it is necessary to consider factors that may significantly interfere with the observed results. For example, in rats, fasting increases APAP hepatotoxicity by depressing the glucuronide and sulfate conjugation pathways, resulting in a significantly increased formation of the toxic metabolite NAPQI. This effect has been reported in humans [53,54]. Furthermore, malnutrition reduces hepatic glutathione levels, limiting the ability to metabolize reactive metabolites and worsening intoxication owing to the deficiency of phase II metabolizing enzymes [53]. In our study, the animals received food ad libitum to avoid interference with results related to their nutritional status. In addition, the animals were subjected to a short fasting period of 6 h before APAP administration to ensure gastric emptying and minimize possible interference in drug absorption. Animal models have demonstrated that ethanol acts as a competitive inhibitor of APAP metabolism mediated by cytochrome P450. However, evidence of the inhibition of APAP metabolism and reduction in APAP toxicity due to the concomitant use of ethanol in humans is limited [52,55]. This mechanism has been used to justify the hypothesis that an acute dose of ethanol has protective effects.

Acute ethanol ingestion inhibits the microsomal oxidation of paracetamol by CYP2E1 and alters the redox state, resulting in the reduced synthesis of NAPQI. However, only a few clinical studies have reported the protective effects of acute ethanol ingestion in humans. [56], it was pointed out that a low proportion of patients who consumed ethanol during acute APAP ingestion developed subsequent hepatotoxicity. According to the authors, factors such as the ingestion of smaller amounts of APAP and early arrival at the hospital for treatment may have influenced the results. In a multivariate analysis that considered the declared dosage of APAP ingested by the patient and the interval between ingestion and hospital admission, acute ethanol consumption had no independent effect on the risk of hepatotoxicity. Finally, the authors suggested that there may be a dose threshold for the possible protective effects of acute ethanol consumption against APAP hepatotoxicity. According to [57], the most important factors determining the development of hepatotoxicity are the relative doses and timing of ethanol and APAP ingestion. This is because CYP2E1 is inhibited in the presence of ethanol [52]. A study [58] found that the hepatic ethanol content was significantly modulated by ethanol ingestion. These authors observed that in a group of active drinkers, the hepatic level of CYP2E1 was, on average, twice as high as that in individuals with moderate abstinence (5–10 days). Furthermore, after the cessation of ethanol consumption, the level of CYP2E1 decreased rapidly and appeared to return to lower levels after five days. Hepatic CYP2E1 levels can be increased up to eight-fold by ethanol in rodents, and its activity is increased in association with the consumption of alcoholic beverages in humans [59].

Alcohol dehydrogenase (ADH) catalyzes the conversion of ethanol to acetaldehyde, which is then rapidly converted to acetate by aldehyde dehydrogenase (ALDH). ADH is activated at low ethanol concentrations. However, ethanol oxidation is limited by the amount of ADH present in the liver. This is a saturable pathway; therefore, ADH is unable to sufficiently oxidize all the ethanol present. When the metabolic capacity via ADH is exceeded, ethanol metabolism via the CYP2E1, CYP1A2, and CYP3A4 pathways is predominant [60,61]. In the present study, it was observed that the CE group presented the highest ADH expression values compared to the other groups.

UDP-glucuronosyltransferases (UGTs), which catalyze the glucuronidation of compounds by transferring glucuronic acid from its co-substrate (UDP-glucuronic acid) to other compounds, are of great relevance in phase II metabolic reactions because of their ability to increase hydrophilicity, enabling the excretion of these substances through bile and urine [62,63]. Regarding the expression of UGTs, we found that, in the case of UGT1A1, the CE group presented significantly higher values than the other groups. A study conducted in C57BL/6 mice found that chronic alcohol intake caused an increase in plasma bilirubin levels and induced an increase in the expression of UGT1A1 mRNA and protein, which mediated the detoxification of bilirubin into less toxic substrates for biliary and urinary excretion [64], thus corroborating the data observed here. The enzyme UGT2A1, also involved in the glucuronidation of xenobiotics, although belonging to the same family as UGT1A1, was downregulated in all treatments compared to the control group. However, it is worth noting that the expression of UGT2A1 was also increased in the CE group compared to that in the APAP group, further suggesting that APAP was metabolized in the CE group through a non-toxic route. The increase in the expression of both isoforms in the CE group compared to the APAP group may contribute to the clearance of toxic metabolites. This, in turn, contributed to the reestablishment of the GSH/GSSG ratio observed in the CE group.

Using spectral data of the soluble fraction of the liver proteome, we identified 937 distinct protein groups among the experimental groups. Proteins were uniquely identified in each study group. Another research group observed that in C57BL/6 mice (susceptible to APAP poisoning) treated with APAP (300 mg/kg), a series of proteins were highly expressed compared to SJL lineage mice (resistant to APAP poisoning) undergoing the same treatment. This difference was attributed to the greater susceptibility of the C57BL/6 lineage to APAP-induced liver injury, which caused the selective loss of hepatic proteins, especially mitochondrial proteins, because of APAP overdose [65]. In our study, the CE group showed the highest number of proteins that could not be detected in the other experimental groups. Prolonged ethanol use in C57BL/6 mice can cause significant changes in hepatic proteins related to phosphorylation, including a range of proteins involved in cell death signaling [66]. We found that treatment with APAP, as well as treatments associated with acute or chronic EtOH consumption, promoted positive regulation of a series of proteins mainly related to metabolism, redox status, and cell signaling. This result is likely associated with a compensatory mechanism to maintain cellular functions in response to the stress induced by APAP and/or ethanol overdose.

The liver is an organ that functions as a critical center for numerous physiological processes essential for the maintenance of life. Drug-induced liver injury is an important cause of liver disease. Alcohol–drug interactions have a potential aggravating effect on drug-induced hepatotoxicity. In the present study, the combination of APAP and an acute dose of ethanol resulted in hepatotoxicity similar to that observed in the APAP group. In contrast, chronic ethanol consumption attenuated paracetamol-induced hepatotoxicity. These findings highlight the complexity of the interactions between ethanol and paracetamol in the context of hepatotoxicity and indicate that further studies are needed to elucidate adaptive response mechanisms during chronic ethanol consumption.

## 5. Conclusions

Ethanol consumption modulates liver damage caused by paracetamol overdose differently. While acute ethanol consumption maintains a liver injury profile similar to the hepatotoxicity profile caused by APAP overdose, chronic ethanol consumption appears to reduce this injury, at least at the dose and time evaluated in this study.

## Data Availability

The original contributions presented in this study are included in the article; further inquiries can be directed to the corresponding author/s.

## Supplementary Materials

The following supporting information can be downloaded at: https://www.mdpi.com/article/10.3390/toxics12120857/s1, Figure S1: Venn diagram showing the overlapped identified protein groups between treatments. Figure S2: Compositional analysis of liver proteome in the mice. (A) The dynamic range of identified proteins encompassed five orders of magnitude difference between the most and least abundant liver constituent, as judged by Area Under Curve (AUC). (B) The cumulative abundance plot revealed 15 molecules contributing to 54% of the total ion signal. Figure S3: Protein genes are evaluated by biochemical analyses according to the KEGG database and categorized according to the metabolic pathways in which they act. Table S1: Main differentially most abundant proteins.

## References

[1] Rotundo L., Pyrsopoulos N. (2020). Liver injury induced by paracetamol and challenges associated with intentional and unintentional use. World J. Hepatol..

[2] Ramachandran A., Jaeschke H. (2019). Acetaminophen Hepatotoxicity. Semin Liver Dis..

[3] Tittarelli R., Pellegrini M., Scarpellini M.G., Marinelli E., Bruti V., Di Luca N.M., Busardò F.P., Zaami S. (2017). Hepatotoxicity of paracetamol and related fatalities. Eur. Rev. Med. Pharmacol. Sci..

[4] Lv L., Ren S., Jiang H., Yan R., Chen W., Yan R., Dong J., Shao L., Yu Y. (2024). The oral administration of *Lacticaseibacillus casei* Shirota alleviates acetaminophen-induced liver injury through accelerated acetaminophen metabolism via the liver-gut axis in mice. mSphere.

[5] Bryan A., Pingali P., Faber A., Landry J., Akakpo J.Y., Jaeschke H., Li H., Lee W.S., May L., Patel B. (2024). High dose acetaminophen with concurrent CYP2E1 inhibition has profound anti-cancer activity without liver toxicity. J. Pharmacol. Exp. Ther..

[6] Maideen N.M.P. (2019). Drug Interactions of Acetaminophen (Paracetamol) involving CYP and UGT Enzymes. Eur. J. Med..

[7] Breslow R.A., Dong C., White A. (2015). Prevalence of Alcohol-Interactive Prescription Medication Use Among Current Drinkers: United States, 1999 to 2010. Alcohol Clin. Exp. Res..

[8] Huang D.Q., Mathurin P., Cortez-Pinto H., Loomba R. (2023). Global epidemiology of alcohol-associated cirrhosis and HCC: Trends, projections and risk factors. Nat. Rev. Gastroenterol. Hepatol..

[9] Hyun J., Han J., Lee C., Yoon M., Jung Y. (2021). Pathophysiological Aspects of Alcohol Metabolism in the Liver. Int. J. Mol. Sci..

[10] Contreras-Zentella M.L., Villalobos-García D., Hernández-Muñoz R. (2022). Ethanol Metabolism in the Liver, the Induction of Oxidant Stress, and the Antioxidant Defense System. Antioxidants..

[11] Iturrospe E., da Silva K.M., Robeyns R., van de Lavoir M., Boeckmans J., Vanhaecke T., van Nuijs A.L., Covaci A. (2022). Metabolic Signature of Ethanol-Induced Hepatotoxicity in HepaRG Cells by Liquid Chromatography–Mass Spectrometry-Based Untargeted Metabolomics. J. Proteome Res..

[12] Kumar S., Singla B., Singh A.K., Thomas-Gooch S.M., Zhi K., Singh U.P. (2022). Hepatic, Extrahepatic and Extracellular Vesicle Cytochrome P450 2E1 in Alcohol and Acetaminophen-Mediated Adverse Interactions and Potential Treatment Options. Cells.

[13] Jaber M.A., Ghanim B.Y., Al-Natour M., Arqoub D.A., Abdallah Q., Abdelrazig S., Alkrad J.A., Kim D.H., Qinna N.A. (2023). Potential biomarkers and metabolomics of acetaminophen-induced liver injury during alcohol consumption: A preclinical investigation on C57/BL6 mice. Toxicol. Appl. Pharmacol..

[14] Coelho A.M., Queiroz I.F., Lima W.G., Talvani A., Perucci L.O., de Souza M.O., Costa D.C. (2023). Temporal analysis of paracetamol-induced hepatotoxicity. Drug Chem. Toxicol..

[15] Coelho A.M., Queiroz I.F., Perucci L.O., de Souza M.O., Lima W.G., Talvani A., Costa D.C. (2022). Piperine as Therapeutic Agent in Paracetamol-Induced Hepatotoxicity in Mice. Pharmaceutics.

[16] Bandeira A.C.B., da Silva R.C., Rossoni J.V., Figueiredo V.P., Talvani A., Cangussú S.D., Bezerra F.S., Costa D.C. (2017). Lycopene pretreatment improves hepatotoxicity induced by acetaminophen in C57BL/6 mice. Bioorganic Med. Chem..

[17] Bandeira A.C.B., da Silva T.P., de Araujo G.R., Araujo C.M., da Silva R.C., Lima W.G., Bezerra F.S., Costa D.C. (2017). Lycopene inhibits reactive oxygen species production in SK-Hep-1 cells and attenuates acetaminophen-induced liver injury in C57BL/6 mice. Chem. Biol. Interact..

[18] Koneru M., Sahu B.D., Kumar J.M., Kuncha M., Kadari A., Kilari E.K., Sistla R. (2016). Fisetin protects liver from binge alcohol-induced toxicity by mechanisms including inhibition of matrix metalloproteinases (MMPs) and oxidative stress. J. Funct. Foods.

[19] Khan I., Bhardwaj M., Shukla S., Min S.-H., Choi D.K., Bajpai V.K., Huh Y.S., Kang S.C. (2019). Carvacrol inhibits cytochrome P450 and protects against binge alcohol-induced liver toxicity. Food Chem Toxicol..

[20] Rocha D.F.A., Machado-Junior P.A., Souza A.B.F., Castro T.D.F., Costa G.D.P., Talvani A., Bezerra F.S., Cangussú S.D. (2021). Lycopene Ameliorates Liver Inflammation and Redox Status in Mice Exposed to Long-Term Cigarette Smoke. Biomed. Res. Int..

[21] Machado-Junior P.A., Araújo N.P.S., Souza A.B.F., Castro T.F., Oliveira M., Costa G.P., Matos N.A., Vieira P.M.A., Talvani A., Bezerra F.S. (2020). Protective Effects of Quercetin on Livers from Mice Exposed to Long-Term Cigarette Smoke. Biomed. Res. Int..

[22] Lowry O.H., Rosebrough N.J., Farr A.L., Randall R.J. (1951). Protein measurement with the Folin phenol reagent. J. Biol. Chem..

[23] Hu S., Yao Y., Wei Z.Y., Wang S.X., Wu Y.C., Hu Y., Yang C.C., Min J.L., Li L.Y., Zhou H. (2022). Deletion of p38γ attenuates ethanol consumption- and acetaminophen-induced liver injury in mice through promoting Dlg1. Acta Pharmacol. Sin..

[24] Kim S.H., Choi H.J., Seo H., Kwon D., Yun J., Jung Y.-S. (2021). Downregulation of Glutathione-Mediated Detoxification Capacity by Binge Drinking Aggravates Acetaminophen-Induced Liver Injury through IRE1α ER Stress Signaling. Antioxidants.

[25] Yang K., Huang Z., Wang S., Zhao Z., Yi P., Chen Y., Xiao M., Quan J., Hu X. (2023). The Hepatic Nerves Regulated Inflammatory Effect in the Process of Liver Injury: Is Nerve the Key Treating Target for Liver Inflammation?. Inflammation.

[26] de Freitas Souza B.S., Nascimento R.C., de Oliveira S.A., Vasconcelos J.F., Kaneto C.M., de Carvalho L.F.P.P., Ribeiro-dos-Santos R., Soares M.B.P., de Freitas L.A.R. (2012). Transplantation of bone marrow cells decreases tumor necrosis factor-α production and blood–brain barrier permeability and improves survival in a mouse model of acetaminophen-induced acute liver disease. Cytotherapy.

[27] Shen X.L., Guo Y.N., Lu M.H., Ding K.N., Liang S.S., Mou R.W., Yuan S., He Y.M., Tang L.P. (2023). Acetaminophen-induced hepatotoxicity predominantly via inhibiting Nrf2 antioxidative pathway and activating TLR4-NF-κB-MAPK inflammatory response in mice. Ecotoxicol. Environ. Saf..

[28] Cederbaum A.I. (2015). CYP2E1- and TNFalpha/LPS-Induced Oxidative Stress and MAPK Signaling Pathways in Alcoholic Liver Disease. Curr. Pathobiol. Rep..

[29] Bourdi M., Eiras D.P., Holt M.P., Webster M.R., Reilly T.P., Welch K.D., Pohl L.R. (2007). Role of IL-6 in an IL-10 and IL-4 Double Knockout Mouse Model Uniquely Susceptible to Acetaminophen-Induced Liver Injury. Chem. Res. Toxicol..

[30] Yan S., Yin M. (2007). Protective and Alleviative Effects from 4 Cysteine-Containing Compounds on Ethanol-Induced Acute Liver Injury through Suppression of Oxidation and Inflammation. J. Food Sci..

[31] Bavia L. (2013). The influence of genetic background of C57Bl/6 and A/J mice on the development of acute inflammatory response induced by alcohol. Rev. Soc. Bras. Ciências Animais Laboratório.

[32] Li C., Ming Y., Hong W., Tang Y., Lei X., Li X., Mao Y. (2018). Comparison of hepatic transcriptome profiling between acute liver injury and acute liver failure induced by acetaminophen in mice. Toxicol. Lett..

[33] Maes M., Vinken M., Jaeschke H. (2016). Experimental models of hepatotoxicity related to acute liver failure. Toxicol. Appl. Pharmacol..

[34] Bavia L. (2021). A/J mice are more susceptible than C57BL/6 to acetaminophen-induced hepatotoxicity. J Pharmacol Toxicol Methods..

[35] Wang X., Wu Q., Liu A., Anadón A., Rodríguez J.L., Martínez-Larrañaga M.R., Yuan Z., Martínez M.A. (2017). Paracetamol: Overdose-induced oxidative stress toxicity, metabolism, and protective effects of various compounds in vivo and in vitro. Drug Metab. Rev..

[36] Herath K.H.I.N.M., Bing S.J., Cho J., Kim A., Kim G., Kim J.S., Kim J.B., Doh Y.H., Jee Y. (2018). Sasa quelpaertensis leaves ameliorate alcohol-induced liver injury by attenuating oxidative stress in HepG2 cells and mice. Acta Histochem..

[37] Noh J.R., Kim Y.H., Gang G.T., Hwang J.H., Lee H.S., Ly S.Y., Oh W.K., Song K.S., Lee C.H. (2011). Hepatoprotective effects of chestnut (*Castanea crenata*) inner shell extract against chronic ethanol-induced oxidative stress in C57BL/6 mice. Food Chem. Toxicol..

[38] Smathers R.L., Galligan J.J., Shearn C.T., Fritz K.S., Mercer K., Ronis M., Orlicky D.J., Davidson N.O., Petersen D.R. (2013). Susceptibility of L-FABP^−/−^ mice to oxidative stress in early-stage alcoholic liver. J. Lipid. Res..

[39] Arumugam M.K., Chava S., Perumal S.K., Paal M.C., Rasineni K., Ganesan M., Donohue T.M., Osna N.A., Kharbanda K.K. (2022). Acute ethanol-induced liver injury is prevented by betaine administration. Front. Physiol..

[40] Dobariya P., Xie W., Rao S.P., Xie J., Seelig D.M., Vince R., Lee M.K., More S.S. (2024). Deletion of Glyoxalase 1 Exacerbates Acetaminophen-Induced Hepatotoxicity in Mice. Antioxidants.

[41] Lebedeva E.I., Babenka A.S., Shchastniy A.T. (2023). MMP-9 mRNA Expression and Bridging Fibrosis Progression in Toxic Liver Injury. Acta Naturae.

[42] Ito Y., Abril E.R., Bethea N.W., McCuskey R.S. (2005). Inhibition of Matrix Metalloproteinases Minimizes Hepatic Microvascular Injury in Response to Acetaminophen in Mice. Toxicol. Sci..

[43] McCuskey R.S. (2006). Sinusoidal endothelial cells as an early target for hepatic toxicants. Clin. Hemorheol. Microcirc..

[44] Ito Y., Abril E.R., Bethea N.W., Mccuskey R.S. (2004). Ethanol Binging Enhances Hepatic Microvascular Responses to Acetaminophen in Mice. Microcirculation.

[45] Xiao Q., Zhao Y., Ma L., Piao R. (2022). Orientin reverses acetaminophen-induced acute liver failure by inhibiting oxidative stress and mitochondrial dysfunction. J. Pharmacol. Sci..

[46] Du K., Ramachandran A., Jaeschke H. (2016). Oxidative stress during acetaminophen hepatotoxicity: Sources, pathophysiological role and therapeutic potential. Redox Biol..

[47] Dogaru G., Bulboaca A.E., Gheban D., Boarescu P.M., Rus V., Festila D., Sitar-Taut A.-V., Stanescu I. (2020). Effect of Liposomal Curcumin on Acetaminophen Hepatotoxicity by Down-regulation of Oxidative Stress and Matrix Metalloproteinases. In Vivo.

[48] Lee S.-M., Cho T.-S., Kim D.-J., Cha Y.-N. (1999). Protective effect of ethanol against acetaminophen-induced hepatotoxicity in mice: Role of role of NADH: Quinone reductase. Biochem. Pharmacol..

[49] Sandoval C., Mella L., Godoy K., Adeli K., Farías J. (2022). β-Carotene Increases Activity of Cytochrome P450 2E1 during Ethanol Consumption. Antioxidants.

[50] Draganov P., Durrence H., Cox C., Reuben A. (2000). Alcohol-acetaminophen syndrome. Postgrad. Med..

[51] Walker R.M., McElligott T.F., Power E.M., Massey T.E., Racz W.J. (1983). Increased acetaminophen-induced hepatotoxicity after chronic ethanol consumption in mice. Toxicology.

[52] Schmidt L.E., Dalhoff K., Poulsen H.E. (2002). Acute versus chronic alcohol consumption in acetaminophen-induced hepatotoxicity. Hepatology.

[53] Bray G.P., Mowat C., Muir D.F., Tredger J.M., Williams R. (1991). The Effect of Chronic Alcohol Intake on Prognosis and Outcome in Paracetamol Overdose. Hum. Exp. Toxicol..

[54] Prescott L.F. (2000). Paracetamol, alcohol and the liver. Br. J. Clin. Pharmacol..

[55] McGill M.R., Jaeschke H. (2013). Metabolism and Disposition of Acetaminophen: Recent Advances in Relation to Hepatotoxicity and Diagnosis. Pharm. Res..

[56] Waring W.S., Stephen A.F., Malkowska A.M., Robinson O.D. (2008). Acute Ethanol Coingestion Confers a Lower Risk of Hepatotoxicity after Deliberate Acetaminophen Overdose. Acad. Emerg. Med..

[57] Bromer M.Q., Black M. (2003). Acetaminophen hepatotoxicity. Clin. Liver Dis..

[58] Perrot N., Nalpas B., Yang C.S., Beaune P.H. (1989). Modulation of cytochrome P450 isozymes in human liver, by ethanol and drug intake. Eur. J. Clin. Investig..

[59] Liangpunsakul S., Kolwankar D., Pinto A., Gorski J.C., Hall S.D., Chalasani N. (2005). Activity of CYP2E1 and CYP3A enzymes in adults with moderate alcohol consumption: A comparison with nonalcoholics. Hepatology.

[60] Wang X., Yang Y., Chen Y., Duan Y., Han J., Yang X. (2022). MEK1/2 inhibitors induce class I alcohol dehydrogenase (ADH1) expression by regulating farnesoid X receptor in hepatic cell lines and C57BL/6J mouse. Mol. Biol. Rep..

[61] Hedgpeth B., Missall R., Bambaci A., Smolen M., Yavuz S., Cottrell J., Chu T., Chang S.L. (2019). A Review of Bioinformatics Tools to Understand Acetaminophen-Alcohol Interaction. Medicines.

[62] Kutsuno Y., Itoh T., Tukey R.H., Fujiwara R. (2014). Glucuronidation of Drugs and Drug-Induced Toxicity in Humanized *UDP-Glucuronosyltransferase 1* Mice. Drug Metab. Dispos..

[63] Shiratani H., Katoh M., Nakajima M., Yokoi T. (2008). Species Differences in UDP-Glucuronosyltransferase Activities in Mice and Rats. Drug Metab. Dispos..

[64] Wang X., Zheng L., Wu J., Tang B., Zhang M., Zhu D., Lin X. (2017). Constitutive androstane receptor activation promotes bilirubin clearance in a murine model of alcoholic liver disease. Mol. Med. Rep..

[65] Welch K.D., Wen B., Goodlett D.R., Yi E.C., Lee H., Reilly T.P., Nelson S.D., Pohl L.R. (2005). Proteomic Identification of Potential Susceptibility Factors in Drug-Induced Liver Disease. Chem. Res. Toxicol..

[66] Singh V., Huang E., Pathak V., Willard B.B., Allende D.S., Nagy L.E. (2022). Phosphoproteomics identifies pathways underlying the role of receptor-interaction protein kinase 3 in alcohol-associated liver disease and uncovers apoptosis signal-regulating kinase 1 as a target. Hepatol. Commun..

