# Supplementary material for: Modulation of Paracetamol-Induced Hepatotoxicity by Acute and Chronic Ethanol Consumption in Mice: A Study Pilot
Source: Toxics. 2024 Nov 27;12(12):857. doi: 10.3390/toxics12120857 (PMC11679532; doi:10.3390/toxics12120857)
Supplement: Supplementary file 1 [file toxics-12-00857-s001.zip › toxics-3329255-supplementary.pdf]

Table S1. Main differentially most abundant proteins.

Acession	Description	Cover (%)	Molecular mass (Da)
Q8C196|CPSM_MOUSE	Carbamoyl-phosphate synthase [ammonia] mitochondrial.	79	164,618
P07724|ALBU_MOUSE	Albumin	80	68,693
P19157|GSTP1_MOUSE	Glutathione S-transferase P 1	82	23,609
P12710|FABPL_MOUSE	Fatty acid-binding protein liver	72	14,246
P47738|ALDH2_MOUSE	Aldehyde dehydrogenase mitochondrial	67	56,538
P16015|CAH3_MOUSE	Carbonic anhydrase 3	86	29,366
Q8R0Y6|AL1L1_MOUSE	Cytosolic 10-formyltetrahydrofolate dehydrogenase	73	98,709
Q91Y97|ALDOB_MOUSE	Fructose-bisphosphate aldolase B	87	39,507
tr|A8DUK4|A8DUK4_MOUSE	Beta-globin	95	15,748
P16460|ASSY_MOUSE	Argininosuccinate synthase	65	46,584
O35490|BHMT1_MOUSE	Betaine--homocysteine S-methyltransferase 1	80	45,021
P19096|FAS_MOUSE	Fatty acid synthase	48	272,428
tr|A0A0R4J135|A0A0R4J135_MOUSE	Methanethiol oxidase	69	52,628
tr|A2BIN1|A2BIN1_MOUSE	Major urinary protein	79	20,682
P02088|HBB1_MOUSE	Hemoglobin subunit beta-1	80	15,840

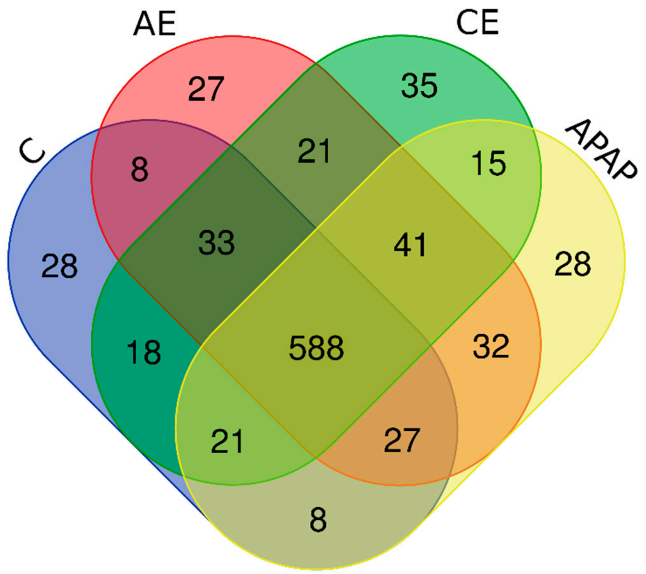

Figure S1. Venn diagram showing the overlapped identified protein groups between treatments.

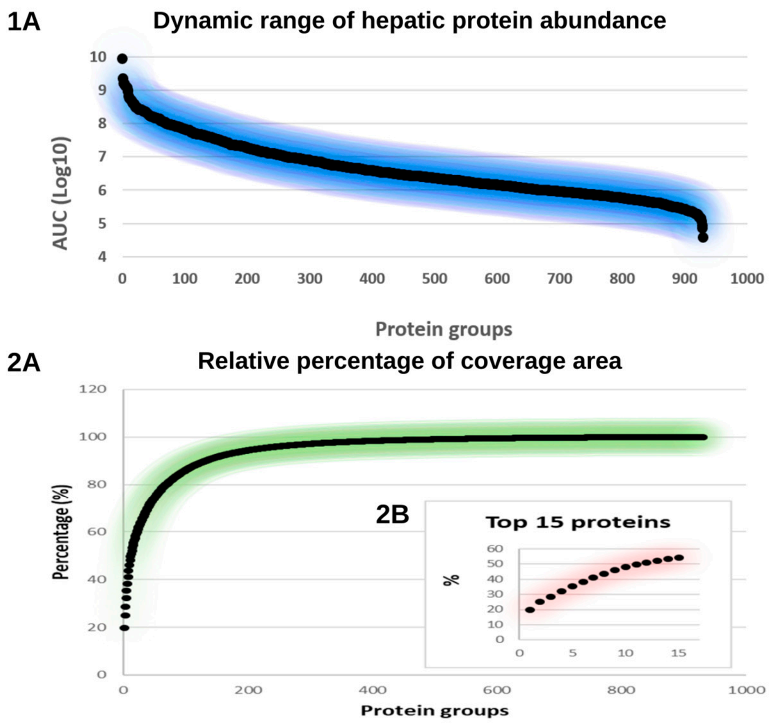

Figure S2. Compositional analysis of liver proteome in the mice. (A) The dynamic range of identified proteins encompassed five orders of magnitude difference between the most and least abundant liver constituent, as judged by Area Under Curve (AUC). (B) The cumulative abundance plot revealed 15 molecules contributing to 54% of the total ion signal.

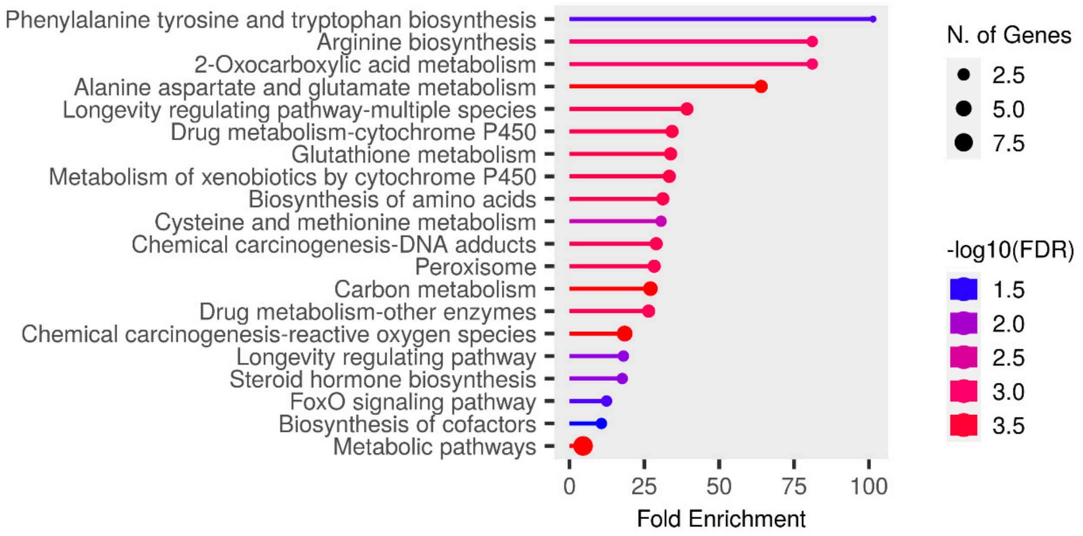

Figure S3. Protein genes are evaluated by biochemical analyses according to the KEGG database and categorized according to the metabolic pathways in which they act.
